# Pluripotent-Stem-Cell-Derived Hepatic Cells: Hepatocytes and Organoids for Liver Therapy and Regeneration

**DOI:** 10.3390/cells9020420

**Published:** 2020-02-12

**Authors:** Antonietta Messina, Eléanor Luce, Marwa Hussein, Anne Dubart-Kupperschmitt

**Affiliations:** 1 INSERM unité mixte de recherche (UMR_S) 1193, F-94800 Villejuif, France; antonietta.messina@inserm.fr (A.M.);; 2 UMR_S 1193, Université Paris-Sud/Paris-Saclay, F-94800 Villejuif, France; 3 Département Hospitalo-Universitaire (DHU) Hépatinov, F-94800 Villejuif, France

**Keywords:** liver regeneration, human pluripotent stem cells, hepatocyte-like cells, cell transplantation, liver organoids, cell therapy, bio artificial liver devices, liver bio-fabrication, organ bioengineering

## Abstract

The liver is a very complex organ that ensures numerous functions; it is thus susceptible to multiple types of damage and dysfunction. Since 1983, orthotopic liver transplantation (OLT) has been considered the only medical solution available to patients when most of their liver function is lost. Unfortunately, the number of patients waiting for OLT is worryingly increasing, and extracorporeal liver support devices are not yet able to counteract the problem. In this review, the current and expected methodologies in liver regeneration are briefly analyzed. In particular, human pluripotent stem cells (hPSCs) as a source of hepatic cells for liver therapy and regeneration are discussed. Principles of hPSC differentiation into hepatocytes are explored, along with the current limitations that have led to the development of 3D culture systems and organoid production. Expected applications of these organoids are discussed with particular attention paid to bio artificial liver (BAL) devices and liver bio-fabrication.

## 1. Introduction

**Figure 1:**
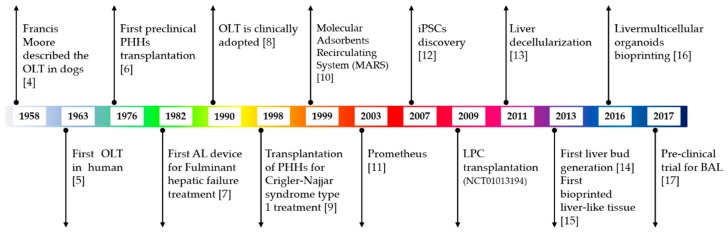
Schematic timeline of liver therapy and regeneration approaches and techniques [4,5,6,7,8,9,10,11,12,13,14,15,16,17]. OLT: orthotopic liver transplantation; PHHs: primary human hepatocytes; AL: artificial liver; iPSCs: induced pluripotent stem cells; LPC: liver progenitor cells; BAL: bio artificial liver.

The liver is one of the most complex organs in the human body in terms of quantity and variety of functions. Defined as an exocrine and endocrine gland and as an organ, it is connected to the digestive system and performs numerous functions useful not only for the digestion of food but also for the defense of the body and the elimination of toxic substances. It is composed of different cell types including, at least, hepatocytes, biliary epithelial cells (cholangiocytes), stellate cells, Kupffer cells, and liver sinusoidal endothelial cells. Each of these cell types possesses unique functions, and their cooperation regulates hepatic function at multiple levels. The hepatocytes, in particular, represent 80% of the adult organ mass and perform almost all the functions related to its metabolic activity. Due to their high regeneration ability, these cells allow for the repair of damaged areas of the tissue, for example after restricted injury or surgery. However, the liver can indeed be damaged by viral infections and inherited genetic diseases, but also by an unbalanced life style, an excess of fat in the diet, unregulated alcohol consumption, smoking, drug use, and excess of medication, all inevitably leading to its dysfunction. Long-term damage can result in the loss of hepatocyte functions, which impacts liver regeneration ability. Liver damage may evolve as acute or chronic liver failure. Acute liver failure (ALF) occurs rapidly and can be due to any of the following: viral infections, such as hepatitis B (5%) or C (12%); drug overdose or individual toxicity of medications (2.4%); metabolic disorders, such as Wilson’s disease (1%); autoimmune diseases (2%); and toxin exposure (4.4%). Chronic liver failure (CLF) is a life-threatening emergency that passes through at least four stages: (i) inflammation; (ii) fibrosis, when healthy tissue in inflamed liver is replaced by scar tissue; (iii) cirrhosis, in which scars can prevent some of the liver functions; and (iv) end-stage liver disease (ESLD) and/or cancer. CLF is generally the result of hepatitis B or C infection (22%), alcohol-related liver diseases (19%), nonalcoholic fatty liver disease, genetic or autoimmune hepatitis (6%), or diseases that affect the bile duct system (14%). Antiviral medications and immune suppressing medications are available today to treat early stages of liver failure; nonetheless, cirrhosis is often not reversible and can be only slowed or stopped. Unfortunately, when ESLD is reached, the loss of liver functions is too important, and decompensation in other organs and systems arises, including hepatic encephalopathy, variceal bleeding, kidney impairment, ascites, and other lung issues [1,2]. In these cases, the only curative treatment is liver transplantation [3]. It is well-known that the principal issue in liver transplantation is the shortage of donors. In this review, we present the current available methodologies in liver regeneration and the new tools developed during the last decade (Figure 1), with the aim to discuss their advantages and weaknesses. In particular, special attention is given to liver organoids, 3D structures generally obtained after differentiation of human pluripotent stem cells (hPSCs) and able to reproduce at least one of the native organ functions. Despite the promising results obtained both in vitro and in pre-clinical trials, improvements still need to be done on hPSC-derived cells and organoids. However, physicians and researchers are counting on these new tools for improving organ transplantation and for the development of new therapies, with a special focus on personalized medicine.

## 2. Liver Therapy and Regeneration Approaches: Pros and Cons

### 2.1. Orthotopic Liver Transplantation (OLT)

Since 1983, orthotopic liver transplantation (OLT) has been considered the only medical solution available to patients with liver failure. Most transplants involve the whole organ; however, partial liver transplantation has been performed with increasing frequency in recent years, potentially allowing for the treatment of multiple patients from one donor. Interestingly, it has been recorded that the age of transplanted patients has increased consistently in the last decade all over Europe as well as the age of the donors (30% donors are now over 60 years old) [1]. Generally, the outcomes for OLT are very good, with 88% of patients surviving for at least one year after the surgery and 73% for five years [1]. Nevertheless, between 1% and 5% of new transplants result in poor functionality of the transplanted liver, leading to 7% mortality. Moreover, many complications related to the surgery may occur, such as hepatic artery thrombosis (2–5%), biliary complications (15%), and/or infections. Another significant impairment is organ rejection (25–50%), occurring within the first year after transplantation with the highest risk period windowed within the first four to six weeks, even if immunosuppressive treatment is constantly optimized to prevent this tremendous drawback. Unfortunately, only 8250 and 7000 liver transplantations were performed in the US and Europe, respectively, out of the nearly 15,000 patients waiting for a graft in 2017, with numbers still worryingly increasing in both regions, with an increasing number of deaths of patients on the waiting list [1,18]. In order to support patients’ liver functions until OLT is available, extracorporeal liver support devices are currently used.

### 2.2. Artificial Liver Devices

Commonly called an artificial liver (AL), the clinically approved molecular adsorbents recirculating system (MARS) from Gambro and the fractionated plasma separation and adsorption (FPSA), commercialized as Prometheus, are devices able to take over the liver detoxification functions while a patient’s liver eventually recovers through regeneration. However, a major problem still remains, as these devices are basically constituted of membrane separation columns (e.g., charcoal, anion-exchange, or cation-exchange resins) that remove toxins and regenerate plasma only partially on a molecular weight cut-off basis. Eventually, the liver regeneration improvement is recorded only for patients with a simple medical history; therefore, for more complex clinical profiles, the risk of deterioration of the patient’s condition remains high [7,19,20]. Indeed, conventional dialysis techniques, such as hemofiltration, hemodialysis, and hemodiafiltration, are in charge of the removal of the low-molecular-weight and water-soluble metabolites; nevertheless, most of the toxins that accumulate in the plasma of patients with liver insufficiency are protein-bounded, greatly reducing the dialysis efficacy. Bio artificial liver (BAL) devices hold the promise of fulfilling enzymatic detoxification, biotransformation, and protein synthesis other than detoxification. Indeed, BALs are generally incorporated with a bioreactor containing a large number of hepatocytes that are aimed at offsetting a patient’s damaged liver functions. Cell lines such as HepG2, Huh7, and HepaRG and immortalized human hepatocytes have been used in pre-clinical trials, showing improvement in pathophysiological parameters in animal models of liver failure [21]. However, their use is not appropriate in clinics, as they come from liver tumoral tissues [22]. Xenogenic hepatocytes were also tested in in vitro studies and some pilot clinical trials such as porcine hepatocytes. They have in fact the advantage of being available in large quantities and obtainable upon request. However, their clinical use is now prohibited due to the immunological problems associated with the production of xenogenic proteins, and to the risk of zoonosis transmission [23]. 

### 2.3. Cell Therapy: Transplantation of Isolated Cell

Over the last thirty years, innovative therapeutic approaches have been proposed as substitutes to organ transplantation. All together defined as cell therapy, they rely on supplying healthy and functional cells for supporting the defaulting functions of the diseased organ. Two categories of cell therapy can be distinguished: (i) the first consists in the injection of an isolated cell suspension, (ii) the second one in grafting bioengineered products (see Section 2.4). Despite restrictions and caveats being still important, both of them are very promising, and interesting results have been reported in many pre-clinical trials [24,25].

In many aspects, cell transplantation shows numerous advantages with respect to the OLT: (i) hepatocytes can be derived from livers that are not suitable for transplantation, (ii) freshly isolated cells can be used to treat one or more patients, and (iii) with respect to OLT, cell injection is far less invasive, which allows for multiple consecutive injections. Hepatocyte transplantation potentially may slow or even reverse liver degeneration process as a consequence of the cellular ability to replace the patient’s damaged hepatocytes and to stimulate the regeneration and repair mechanisms of the surrounding diseased tissue. As a consequence, the restoration of the basal liver functions is possible. In 1998, after pre-clinical trials of human hepatocyte transplantation were carried out on mice and rats [26,27], the New England Journal of Medicine reported a successful transplantation in a 10-year-old girl affected by Crigler–Najjar syndrome type I and suffering from a severe unconjugated hyperbilirubinemia of cadaveric primary human hepatocytes. The hepatocytes were safely infused through the portal vein, demonstrating that this procedure has a lower surgical risk with respect to OLT. The survival of the transplanted cells for more than 11 months was recorded and resulted in the partial correction of the metabolic disorder by allowing the secretion of bilirubin glucuronides in the same proportions as those in normal bile [9]. Since then, other important results have been achieved in treating patients with acute liver failure and genetic disorders [28,29,30,31]. Patients with inborn liver-based metabolic disorders are good candidates for cell transplantation, presenting only defects in enzymes or transport proteins for which even partial correction will significantly improve a patient’s condition. Successful clinical trials have been reported in treating liver-based metabolic disorders in pediatric patients, and numerous studies are currently being carried out in order to improve cell availability and safety as well as medical procedures [32,33]. Nevertheless, all these studies allowed for the identification of some critical parameters for the optimal organ repopulation by transplanted cells and the anticipated clinical outcome. When adult or more complex clinical conditions must be treated, a preconditioning of the resident liver needs to be carried out. Pre-clinical studies demonstrated that portal vein partial embolization (PVPE) or partial hepatectomy are procedures that allow a nearly 50% improvement in the transplanted cell engraftment [34,35]. However, these clinical settings are not easily transferable in clinical trials [6], and an alternative site of transplantation and other preconditioning approaches such as the use of reversible PVPE [35,36,37] and the irradiation of the native liver need to be further investigated [6,38].

If hepatocyte transplantation were to achieve its full potential, OLT could be potentially used only for treating the most serious cases of liver failure. However, the critical shortage of donors precludes recovering the number of hepatocytes needed for transplantation to result in a therapeutic benefit. Indeed, despite new procedures having been proposed to improve the standard method for isolating hepatocytes from human liver tissue [39], many problems still remain. Once isolated, cells rapidly lose their phenotypic characteristics and functions [40]. This may be due in part to the loss of tissue architecture (loss of cell–cell and cell–matrix interactions) and to the endotoxins contained in the collagenase used for their isolation. Furthermore, they possess no proliferative capacity in vitro and thus cannot be amplified before transplantation. A great effort has been made in the realization of the frozen banks of cells to increase clinical availability, but unfortunately the loss of functions and morphological characteristics after thawing still make their use very difficult. Only assuring maximal functional cell delivery with the best engraftment will constitute a reliable alternative to OLT. As a consequence, and beside the necessary technical improvements, cell transplantation requires finding alternative functional hepatic cell sources (see Section 3) and/or optimizing cell delivery through bioengineered approaches. 

### 2.4. Cell Therapy: Bioengineering Approaches

In view of the enormous progress recorded in the past decades, bioengineering approaches may provide suitable alternatives to further improve liver regeneration and therapy [41]. One of the new approaches proposed in this frame of liver cell therapy is using liver spheroids/organoids as transplantable units. These miniaturized and simplified versions of an organ produced in vitro have been able to sustain cell activity and long-term functions in vitro and in in vivo pre-clinical trials [23]. However, new concepts are still under investigation. Moreover, in vitro and in vivo studies showed that the use of a biomimetic environment that envelops the cells before transplantation can enhance survival and functionality of the engrafted cells [42]. This led to liver regeneration and repair in CCl_4_-treated mice, suggesting that implantable devices could eventually represent a new strategy for liver regenerative medicine in the treatment of both acquired pathologies and genetic disorders [43]. Biocompatible polymers such as collagen, gelatin, and alginate could represent a good choice for improving cell transplantation [44,45]. In the following sections, some of the new bioengineering approaches currently used in liver therapy and medicine regeneration are discussed. 

#### 2.4.1. Liver Organoids

Hepatocytes in 3D configuration may represent a promising tool for implementing liver cell therapy. Therefore, in the next section, a rapid overview on the 3D culture evolution is presented alongside the current liver organoid applications. Indeed, a monolayer culture at the bottom of a culture plate (2D) is not very representative of physiological conditions, even if 2D co-cultures have allowed for the discovery of most of the physiological mechanisms known today. To overcome this problem, some teams have developed co-culture, or 3-dimensional (3D) culture, approaches. These 3D approaches allow for the generation of in vitro cellular structures that, with minimal external cues, mimic fetal or adult organ-like tissue, exhibiting, even if partially, a complex level of native tissue organization and functions [46]. Called “organoids,” these cellular structures are derived from the differentiation of embryonic stem cells (ESCs) and induced pluripotent stem cells (iPSCs) or adult stem cells in a 3D environment. In such particular conditions, tissue-like arrangements, compartmentalization, and functionality are results of the cell’s ability to aggregate, sort, and re-organize to form a 3D tissue mass, even when randomly dispersed [47]. The self-organization process does not match real organogenesis in detail; however, if the in vitro optimal physico-chemical cues are provided, they could potentially result in a miniature reproduction of the organ of interest. This is the reason why liver organoids are becoming more important in the regenerative medicine field and are considered biological blocks for organ replacement, disease modeling, toxicological studies, and drug discovery [48]. However, organoid generation is not so easy. Intestinal organoids or hepatic organoids, for instance, are obtained through the subtle choice of concentrations of specific growth factors common to both organogenesis processes [49,50]. Indeed, the very same organoid can shift from one tissue specification to another if conditions change during its generation process [51]. Choosing and adjusting a certain set of parameters in detail is therefore important. In order to control these processes, it is mandatory to (i) choose and control cell numbers and density for the dissociation–aggregation phase, (ii) choose the best culture conditions and the correct growth factor cocktails to specifically guide the differentiation process, (iii) provide, if necessary, pre-defined extrinsic forces to enhance cell–cell interactions, by means of avoiding or adding extracellular matrix (ECM) and/or matrices, and (iv) choose the most adapted engineered geometry to support cell and organoid availability, such as micropatterning, microwells, and microfluid dynamics [46]. 

*Organoid generation*. Simply speaking, all organoid generation protocols start with a process defined as a “dissociation–aggregation” approach in which cells are dissociated and then seeded in a specific and controlled environment [47]. Some of the available and well-established methods for generating organoids are listed in Table 1. PSCs are dissociated and either used to generate embryoid bodies (EBs) in suspension or seeded as a homogeneous sheet under differentiation conditions. EBs are aggregates of PSCs that spontaneously generate the three germ lineages in suspension. Cell differentiation and morphogenesis result in microtissues that are similar to embryonic tissue structures in which the appearance of both epithelial- and mesenchymal-like cell populations can be observed, as well as the appearance of markers associated with the epithelial-mesenchymal transition (EMT) and the ectoderm-derived neural lineages. Mimicking essentially the embryological development in vitro, EBs are very useful in many frames of biology and regenerative medicine. Nevertheless, because of their high structural complexity, EBs present a drawback: the directed differentiation towards a specific cell lineage is very challenging and many studies are currently carried out in order to improve the control of cell differentiation and fate [52]. When PSCs are seeded as a monolayer, the risk of uncontrolled development or cell specification is kept under control. Indeed, the addition of growth factors and morphogens as well as the extracellular microenvironment can precisely direct the differentiation towards a pure cell population generally constituted of progenitors. These cells can be harvested and later used, either alone or with other cell types, to generate organoids with higher functionality in a 3D environment. 

The first keystone of such an approach was the work of Takebe and colleagues, who demonstrated that the generation of a human liver bud containing endothelial cells was possible. Moreover, they demonstrated that, once transplanted, such a bud was able to develop an endothelial network within only 2 days [14,51]. Another important parameter is whether or not the self-assembling process will be regulated through external or intrinsic forces. Using a matrix reproducing the native tissue ECM or a scaffold with controlled physico-chemical and mechanical characteristics can further define the self-organization of the organoids and their level of functionality. Micro-patterned culture supports, micro-fluidized systems, or scaffolds defined in stiffness and porosity are widely used, as well as decellularized natural scaffolds. In this context, hydrogels appeared surprisingly suitable showing a high degree of structural flexibility and able to compress organoids to guide their formation within specific features such as dimensions and shape, while remodeling themselves during the process [46,53]. On the other hand, in the regenerative medicine field it is essential to minimize the risk of rejection and tissue failure, which are common with scaffolds. A scaffold-free approach can theoretically overcome the problems without interfering with the control of cell differentiation and self-organization [54]. 

*Liver organoid current applications.* Organoids have been successfully used as new candidates for many applications such as disease modeling and drug screening. Many disease models have been developed, and through them pathologies such as familial hypercholesterolemia, Crigler Najjar, Hemophilia A, Wilson’s disease, α1 antitrypsin deficiency, liver fibrosis, NASH, NAFLD, and acquired diseases due to toxin products have been modeled and better characterized, leading to a better knowledge and withdrawal of existing drugs and to the identification of new ones [55,56,57]. In fact, only 10% of drug screening clinical trials, non-withdrawn during phases I and II, succeed to the last phase and can be proposed as new candidates for medical treatment. Sixty-six percent of these molecules show a lack in efficacy, and 21% of them lead to safety issues [58]. Being able to express more cytochrome P450 and other phase II enzyme activities, organoids have shown a better response to apoptotic drugs and can correctly metabolize molecules such as rifampicin, omeprazole, phenobarbital, and paracetamol [59,60], allowing for the discovery of several drug-adverse effects on the human liver [61,62]. Moreover, these 3D constructs can reproduce typical polymorphisms between individuals, allowing for further progress in personalized therapy development [63]. The organoid is now considered the most appropriate tool for evaluating drug efficacy, identifying mechanisms underlying certain diseases, and screening drugs before animal testing [64,65]. Platforms using organoids as a counterpart to animal models should help researchers to collect more information from both models and to compare them, improving the predictions for clinical outcomes. Great interest has arisen also around the organ-on-chip systems, in which microfluidic perfusion can generate in vitro physiological features that further improve the insight into drug metabolism and disease modeling. Details such as first-pass metabolism and drug clearance are surely better mimicked in perfusable chip systems rather than in 2D or 3D suspension cultures [66,67].

#### 2.4.2. Bio Artificial Liver (BAL) Devices

As previously described, a cell-housing bioreactor was conceived to improve AL devices in order to perform hepatic functions such as oxidative detoxification, biotransformation, excretion, and synthesis. Since then, clinical investigations of BAL have been proposed, and many reviews have been published on the historical and functional evolution of these systems since the first successful devices used in 1987 [7]. However, culturing cells in a bioreactor means that the cells are eventually exposed to (i) a continuous flow and consequently to shear stress and mechanical forces that can eventually result in damage and loss of viability, (ii) toxins present in the plasma that need to be treated, and (iii) waste products derived from detoxification and cellular metabolism, including bile. A major problem affecting BAL improvement and its clinical translation is finding an expandable source of functional hepatocytes that perfectly combine availability, performance, and associated risk, since primary human hepatocytes (PHHs), in spite of being the gold standard, have strong limitations in terms of availability and quality [68]. Hepatocytes derived from the in vitro differentiation of pluripotent stem cells have been proposed as new cell sources for BALs in the form of either cell suspensions or organoids. Selden and colleagues in 2017 designed and tested a clinical-scale BAL designed to meet all requirements for the manufacture of advanced therapy medicinal products (ATMPs) that are mandatory for clinical trial acceptance. After culturing human hepatoblastoma cells as three-dimensional organoids in a fluidized bed bioreactor, the complete control of nutrient provision was recorded, and good phenotypic liver functions were achieved. In order to further validate the device, a porcine model of severe liver failure was used for pre-clinical testing. All animals underwent surgical ischemia and were then branched to the BAL. A significant improvement in animals’ general conditions was detected. Using the same approach, the development of BALs that host human liver organoids can finally be achieved in the near future [17]. However, some final challenges must be faced. First, differentiated hepatocytes not only need to be preserved from shear stress and toxin accumulation, but they also need to be cultured in an environment that is able to sustain their acquired functionality. Encapsulated cells, as for the transplantation procedure, are today used to improve BALs. Both rodent and pig models have demonstrated a successful recovery from liver failure after treatment. Second, as previously stated, many circulating products can impair BAL functionality. In particular, both the bile and bile salts accumulated in the patient’s plasma and the ones produced by the biological component of the BAL constitute a real issue. Bile formation and excretion is a complex mechanism involving an entero-hepatic recirculation. When the bile circuit is not successfully managed, its accumulation into the hepatocytes leads to cell damage and eventually necrosis [69]. Bile salts, drug metabolites, bilirubin, and cholesterol can eventually saturate the BAL and flow back into the patient’s plasma. The challenge of managing the bile collection and removal within the BAL is tremendous. Even though many efforts have been made to obtain biliary structures in vitro [70,71], their incorporation into a BAL system is highly complex. Up to now, only chemical–physical detoxing devices have been used and tested, demonstrating that bilirubin and phase II metabolites can be successfully removed, increasing plasma detoxification levels and patient remission.

#### 2.4.3. Decellularized/Cellularized Liver Scaffolds

The bioengineering of the whole liver has always been clinically attractive because of its therapeutic potential for liver transplantation. A scaffold-based strategy that relies on the use of biomaterials to create a temporary structure able to support all liver cell attachment and proliferation is therefore very attractive and may allow a real improvement in generating 3D tissues readily suitable for transplantation and liver therapy [72,73]. As previously mentioned, despite the increased number of donors in the last decade due to the increasing age, an important part of the livers available is still rejected for OLT. In 2011, Baptista and colleagues reported the generation of vascularized liver organoids using a whole decellularized liver as a scaffold for the co-culture of human umbilical endothelial cells and human fetal liver cells. Using their knowledge on decellularization processes, they developed a new method for obtaining a cell-free liver matrix theoretically perfect to pursuit the project of creating a tissue-engineered liver graft. This process had the advantage of perfectly retaining the most important ECM molecules required for site-specific engraftment and differentiation of fetal liver cells. Without going into detail, decellularization is a process that chemically removes all cells from an organ, revealing the ECM that could be this way used as a scaffold for a new cell reseeding in order to rebuild a functional reproduction of the original organ. Liver progenitors and endothelial cells were perfused through the vasculature and were able to repopulate the scaffold in most of the areas. Hepatocytes obtained in this construct were able to express most of the typical differentiation markers (AFP, CYP2A, and CYP3A), and cholangiocyte-like cells were retraceable to the very same construct [13]. However, cell seeding presented one major deficiency: the delivery of an adequate number of cells into a thick scaffold is particularly difficult and requires the use of different vessels as accessing sites. Thus, cells were delivered selectively to different compartments of the scaffolds, which explains the jeopardized repopulation of the device. Since then, many attempts have been made to overcome the major drawbacks of this new approach in liver regeneration. However, all the animal tests published show an important adverse effect: the poor viability of organoids after transplantation (8 h), which results in thrombosis within the transplanted devices [74]. In spite of the animal test positively carried out, a proof of concept of the usefulness of this new approach in a real clinical trial has yet to be done. 

#### 2.4.4. Liver Biofabrication and Bioprinting

The evolution leading to organoid generation has improved medical research. However, one final challenge remains: the possibility of creating a whole liver in vitro. It is well known that statistically only 20% of the liver is constituted by non-parenchymal cells and 80% is hepatocytes. Reproducing this proportion between cells in vitro is possible; however, this is not enough to obtain a real mimic of the organ. In the normal adult human liver, each functional unit, the lobule, is constituted by hepatocytes, which are arranged in cords (or laminas) converging towards a central vein and delimiting a system of hepatic sinusoids (capillaries) that connect the branches of the portal vein with the centrilobular vein. Alongside at least a branch of the hepatic artery and a bile duct, the hepatic portal vein constitutes the so-called portal triad, and up to six triads delimit the lobule periphery [75]. Indeed, generating a construct where cells are able to mimic such a complex layout has yet to be achieved. However, as already presented in this review, many improvements are already being made, and bioprinting is probably the sole technology that holds today the greatest potential for generating bioengineered livers on large scale through either a scaffold-free or a scaffold-based approach. Cells, matrices, and their complex special configurations can theoretically be printed, ensuring at the same time biological, mechanical, and structural support thanks to the so-called bioink. These purposely made biocompatible materials possess viscosity and physico-chemical properties able to maintain the viability and functionality of cells, even if obtained by differentiation from PSCs. Gelatin, alginate, fibrin, hyaluronan, laminin, collagen, and even agarose can be used, depending on the type of cell/organ desired [76,77]. The 3D tissue and organ building allow for complex and detailed spatial control of the cell deposition. Several different bioprinting techniques have been developed [78,79] such as laser pulses and inkjets, and the ability to transfer cell suspensions and/or more complex structures, such as organoids, into well-defined three-dimensional microscopic patterns could lead to the generation of constructs that closely mimic the native tissue architecture [80,81]. Many efforts have been made in developing deposition processes that potentially do not stress cells to avoid impact cell/organoid survival [82]. The impact on the substrate as a consequence of the deposition velocity, shear stress generated in the capillary tubes used for the deposition [83], heat, and high frequency vibration can damage cells [84]. Up to now, only three bioprinted liver-like tissues have been generated. The first experiments date back to 2013, when Faulkner-Jones and colleagues printed human ESCs (hESCs) using a valve-based printing approach and investigated the post-printed viability and pluripotency of their construct [15]. However, a non-homogeneous cellular differentiation was detected, making it difficult to generate a liver-specific tissue. Two years later, another report was published, and, for the first time, investigation was focused on bioprinted hepatocyte-like cells derived from human iPSCs (hiPSCs). Using a droplet dispensing system, differentiated cells were printed into 24 multi-well plates with a higher survival rate with respect to classic 3D cultures used as controls. Forty layers were successfully generated without influencing the differentiation process or cell viability, and specific markers such as HNF4α, tight-junction 1, and albumin expression were detected after 23 days of culture. The only drawback was that these constructs took longer to complete their maturation with respect to the 3D culture controls [85]. Most recently, a 3D hydrogel-based tri-culture model constituted by hiPSC-derived progenitor cells, human umbilical vascular endothelial cells (HUVECs), and adipose-derived stem cells was constructed and described in 2016. Co-culture of liver progenitor cells, mesenchymal stem cells (MSCs), and endothelial cells were precisely patterned by means of a digital light 3D printer in order to reproduce the vascular system and improve hepatocyte maturation. Cells were encapsulated in a hydrogel and printed in a microscale hexagonal architecture. This process sustained a higher level of albumin production compared with a 2D monolayer culture and a 3D encapsulated-only model, showing both phenotypic and functional enhancements over a period of weeks and in terms of urea synthesis and expression of specific hepatic markers [16].

The different approaches currently used for liver therapy and regeneration that have been detailed above are recapitulated in Table 2 and compared in terms of benefits and drawbacks.

## 3. Cell Sources for Hepatocyte Transplantation and Liver Repair

### 3.1. Primary Human Hepatocytes (PHHs)

As described above (see Section 2.3.), despite still being considered the gold standard in research, PHHs present drawbacks that greatly limit their use in clinical applications. Therefore, defining and validating new sources for functional hepatic cell supply is mandatory. 

### 3.2. Fetal Liver Progenitors (FLPs)

Fetal liver progenitors (FLPs) have many advantages compared to adult hepatocytes; they are bipotent cells in vitro, so they can differentiate into hepatocytes and cholangiocytes. They are very proliferative when major hepatic regeneration is necessary. It has been demonstrated that early human and non-human primate fetal hepatoblasts are able to engraft, proliferate, and mature in immunodeficient mouse livers, repopulating 10% of the organ without conditioning the donor [86,87]. More recently, FLPs isolated from human fetal livers have been successfully transplanted into cirrhotic immune-permissive mice. Thirty-six weeks after surgery, cell engraftment and differentiation into functional human hepatocytes in the mouse were detected. Moreover, it has been suggested that FLPs can also transdifferentiate into functional human endothelial cells with no evidence of neoplasia observed within nine months after transplantation. However, the contamination by endothelial cells in the transplanted LFP cell population was not formally excluded [88]. Being less apoptotic and immunogenic than adult hepatocytes, their smaller size allows for an easier intraportal injection and dispersion compared with primary cells [89]. A clinical trial carried out (2009–2015) between ISMETT, Palermo, and the University of Pittsburgh (UPMC-USA) resulted in the transplantation of liver progenitor cells isolated from the human fetal liver tissue to improve conventional liver therapy using proliferative cells able to develop a suitable liver mass to support the patient decompensated liver. The aim of the trial was the investigation of the possibility to generate an ectopic liver system in the spleen through arterial injection of non-purified and non-selected fetal liver cells isolated from between the 16th and 26th week of gestation incannulated by the femoral artery. Between 5 and 10 × 10^8^ cells were transplanted and up to two injections were carried out on the same patients (18–70 years old). No particular adverse effects and a slight transitory improvement in patients’ conditions were recorded. (ClinicalTrials.gov, Identifier: NCT01013194). 

### 3.3. Adult Human Liver Stem Cells (AdHLSCs)

Adult human liver stem cells (AdHLSCs) can also be considered in the frame of cell transplantation. Several studies have suggested the presence of stem cells in the adult normal human liver. A population positive to mesenchymal stem cell markers (CD29, CD73, CD44, and CD90), while expressing albumin and α-fetoprotein, was described in the early 2000s, indicating the possibility that a stem cell population may be present in the adult human liver [90,91,92]. However, this issue remains controversial, as AdHLSCs possess several advantages over adult hepatocytes or fetal progenitors as they are able to proliferate in vitro [93,94]. Moreover, their use does not entail the ethical complications related to the use of fetal cells. It has been shown that these cells also exhibit a propensity for directed differentiation into functional hepatocytes (urea detoxification and glucose synthesis) and a preclinical study reported the absence of tumorigenesis despite the long-term culture before transplantation [95]. 

### 3.4. Hematopoietic Stem Cells (HSCs) and Mesenchymal Stem Cells (MSCs)

As the liver is a hematopoietic organ during the first stage of embryogenesis, representing a major source of erythrocytes in the first trimester of pregnancy, hematopoietic stem cells (HSCs) and mesenchymal stem cells (MSCs) have been proposed as an alternative to hepatocytes for cell transplantation [96]. HSCs appear to have the potential to contribute to hepatic regeneration in humans, as demonstrated by the presence of hepatocytes carrying a Y chromosome in a female patient who received a bone marrow transplant derived from a male [22], but this is most likely due to cell fusion rather than by the transdifferentiation of HSCs into hepatocytes, as shown in a mouse model [97]. Animal studies then confirmed this regeneration capacity in animal models such as mouse [98] and pig [99]. However, the mechanism of action of this regeneration is not fully understood, and some of these studies showed that regeneration is actually due to a fusion of the transplanted cells with the resident hepatocytes of the animal. MSC studies demonstrated that this multipotent cell population plays a role in liver fibrosis when derived from the bone marrow [100] and that the benefits of direct transplantation could be due to their angiogenic properties rather than to their true differentiation potential. Moreover, MSCs are able to immune-modulate the cell response, becoming a new resource for liver regeneration. Unfortunately, the already completed clinical trials show no signs of real improvements in the patient’s recovery, and data are not freely accessible. Indeed, a pronounced debate is ongoing concerning their functional properties, the phenotypic stability once transplanted, and their long-term contribution to tissue homeostasis that has yet to be demonstrated. However, there are high hopes for this approach, and ongoing trials and new recruitments are being registered today worldwide [101]. 

### 3.5. Human Pluripotent Stem Cells (hPSCs)

The great need for alternative renewable sources of human hepatocytes blooms in the use of the hPSCs. Their ability to differentiate into hepatocytes makes them a potentially unlimited source of hepatic cells not only for transplantation and gene therapy but also for the improvement of the temporary support devices for which an additional biological component constituted of functional hepatic cells has been foreseen [19,20]. hPSCs have the ability to undergo self-renewal and to give rise to all cell types after differentiation. Several protocols have been set up to differentiate human embryonic stem cells (hESCs) [102,103] and later human induced pluripotent stem cells (hiPSCs) [104,105,106]. This guided differentiation is based on the different steps of the embryonic development of the liver, mimicked by the addition of growth factors or small molecules in the culture medium. This new approach allows for an almost inexhaustible source of hepatocytes. hiPSCs present the very same unlimited proliferation capacity, plasticity, and pluripotency of the more controversial hESCs, offering a great turning point in disease modeling, drug development, and regenerative medicine. Since they are not derived from human embryos, hiPSCs do not suffer from the same ethical concerns that hESCs do. Moreover, as they can be generated from individual patients, they pave the way to the development of “personalized” medicine. Since Yamanaka and associates’ keystone work in 2007 [12], the advantages of using hiPSCs over hESCs have been explored and demonstrated, particularly concerning in vitro hepatocyte differentiation and maturation [55,106,107]. Generally called hepatocyte-like cells (HLCs), cells generated from hiPSCs show several similarities in morphology, protein expression, and functionality with native hepatocytes. However, a certain degree of immaturity, consistently recorded in all culture systems hitherto developed, represents a major drawback. Though albumin secretion, glycogen synthesis, and detoxification abilities by the CYP450 cohort enzymes are regularly observed, their expression still remains lower than that in PHHs. In addition, other fetal markers and features, such as α-fetoprotein secretion and alcohol dehydrogenase (ADH) inactivity, characterize HLCs [108]. Nevertheless, they have been demonstrated to be able to engraft and mature in animal models [103]. In order to generate HLCs in vitro and use them for any kind of application, it is of major importance to be able to reproduce what happens during embryogenesis and beyond to obtain functional HLCs.

### 3.6. Overview on Embryogenesis and Published Differentiation Protocols for hiPSC Differentiation into HLCs

It is well known that, after fertilization, a segmentation process induces the division of the fertilized egg into non-differentiated cells called blastomeres. These cells further evolve through multiple steps until the three embryonic germ layers are generated: endoderm, mesoderm, and ectoderm. Each one of these layers will subsequently give rise to specific differentiated cell types. The inner layer of the embryo, the endoderm, generates, among many others, digestive organs such as the liver. The hepatic specification of the endoderm results in the generation of liver progenitor cells, the hepatoblasts (HBs). These cells migrate into the septum transversum where they keep proliferating, allowing for the growth of the so-called liver bud. These events occur on embryonic day 9 in mice and day 24 in human development. The formation of this bud is due to the very complex system of signaling and pathways [109,110,111,112]. HBs are bipotent and can either differentiate into hepatocytes or cholangiocytes. The hepatoblast differentiation into hepatocytes is regulated by numerous factors, and the comprehension of molecular mechanisms occurring during liver embryogenesis has contributed to the development of differentiation protocols in vitro [113,114]. Numerous studies on mice have highlighted a complex network of transcription factors necessary for this process, such as members of the hepatocyte nuclear factor family (HNF) including Hnf1α, Hnf1β, Hnf3α, Hnf3β, Hnf3γ, Hnf4α, and Hnf6 [115]. The absence or impairment of one or more of these factors may not impact the hepatic specification but will lead to a blockage of the hepatic progenitor differentiation as well as to a disrupted liver organization [116]. Taking all these pathways into consideration, the first step for HLC generation is to induce hiPSC differentiation into the specific embryonic germ layer from which the precursors arise: the endoderm. In the absence of a self-renewal factor such as FGF2 (fibroblast growth factor 2) in humans or LIF (leukemia inhibitory factor) in mice, pluripotent stem cells (PSCs) spontaneously differentiate in suspension and form aggregates called “embryoid bodies” composed of differentiated cells from the three germ layers. In 2002, Jones et al. took advantage of this phenomenon and showed that the culture of these embryoid bodies allowed for the recovery of hepatocytes after 12 days of culture [117]. A year later, Rambhatla et al. published the first protocol for hESC differentiation in functional hepatocytes [118]. All the protocols developed so far are based on a first step of activin/nodal pathway activation, most of the time by a high concentration of recombinant activin A (100 ng/mL), a member of the TGFβ family. In the most robust protocols, more than 90% of cells expressing CXCR4, a membrane receptor specific to the endoderm, can be obtained. The study by Matsuno et al. showed the importance of choosing the right conditions for differentiation in order to select the most appropriate cell population in the very first days [119]. Once the definitive endoderm is obtained, members of the BMP family, secreted by the septum transversum during embryonic development, as well as FGF2 at high concentrations secreted by the developing heart, are used to specify the hepatic progenitors. The most robust protocols allow for the obtainment of up to 90% of cells expressing both AFP (α-fetoprotein), the fetal form of albumin, and CK19, a cytokeratin that is later expressed during cholangiocyte differentiation but no longer expressed in the hepatocyte lineage. Finally, the last step of the protocol consists in differentiating the bipotent progenitors into the hepatocyte—not the cholangiocyte—lineage and in functionally maturing the differentiated cells. Most of the protocols published to date use several growth factor cocktails, including, for the most commonly used cytokines, hepatocyte growth factor (HGF) and oncostatin M. Nevertheless, some studies have also shown an improvement in differentiation through the use of inhibitors of the notch pathway, such as the compound E [120], or inhibitors of the TGFβ pathway, such as SB431542 [120,121,122]. The use of SB431542 can, however, also lead to undifferentiated hepatoblast proliferation, potentially leading to the emergence of a heterogeneous population composed of both hepatocytes and hepatoblasts. The precise timing of exposition of differentiating cells to such compounds is thus of major importance. Differentiated cells express hepatocyte markers such as HNF4α, albumin, A1AT (α-1-anti-trypsin), and CYP3A7 (fetal form of CYP3A4). A functional study of these cells is essential to ensure their correct differentiation. Among the most studied functions, we mention here albumin synthesis, functions related to energy metabolism, such as glycogen storage assessed by PAS (periodic acid–Schiff) staining, and detoxification functions, such as P450 cytochrome induction by such agents as rifampicin or omeprazole [123]. The sufficient cell differentiation but incomplete maturation in culture can be explained by the fact that the acquisition of most complex hepatocyte functions requires a defined microenvironment that is characterized by spatial organization and by interactions between hepatocytes and with other cell types present in the liver. 

### 3.7. Genetic Integrity

It is mandatory to demonstrate that transplantable cells are nontoxic and otherwise safe. Primary cells such as PHHs, AdLSCs, FLPCs, or MSCs are mostly prone to cell aging DNA damage or immunogenicity, as are the cells in the organ to be transplanted. Thus, they do not present additional risk. If the cells are submitted to an in vitro culture or to cryopreservation, the possible effects of these added steps on the safety of the final product should be limited by the use of validated processes and traceable reagents, as this is already the case for MSC and HSC transplantations. Moreover, results obtained in vitro and from pre-clinical trials have shown the potential of hPSC-based therapy in the treatment of liver pathologies and disorders. However, leaving aside the ethical concerns related to the use of hESCs and derivatives, safety issues regarding hPSC-based therapy are still relevant. Indeed, hPSCs and derived cells, in order to obtain approval for clinical use, need to undergo safety and toxicity studies, especially due to genomic integrity concerns. Up to now, three hypotheses are considered to explain the presence or the acquisition of genetic mutations in hPSCs and derivatives [124]. The first is that pre-existing genetic aberrations/mutations are present in the initial somatic cell population; in that case, the very same mutations can be selected during the reprogramming process by chance or because of selective mechanisms. The second is that mutations can be acquired de novo during the reprogramming process; unfortunately, an optimal reprogramming method with no impact on the cell genome has not yet been established [125]. The third hypothesis is that genetic aberrations can be induced or selected during long-term hPSC culture. How the accumulation and extent of these mutations might influence the function, tumorigenicity, genetic stability, and immunogenicity of therapeutic cell populations is not known. Preclinical studies are able to accurately assess the risk associated with cell therapies. Positron emission tomography (PET) imaging, magnetic resonance imaging (MRI), fluorescence imaging (FLI), and bioluminescence imaging (BLI) are routinely used to evaluate undesired effects [126], but much is still to be done before hPSC derivatives can be routinely used in clinics. However, right now, we can advocate that the genomic integrity of the hPSCs used for the differentiation into therapeutic cells has to be carefully checked and that proper processes to maintain, to amplify, and to differentiate these hPSCs have to be applied [127]. Second, a validated process to totally eliminate undifferentiated hPSCs is mandatory. Indeed, the plasticity that permits the generation of different cell types from differentiating PSCs also makes them difficult to control after in vivo transplantation [128], and by definition they are teratogenic, as a proof of their pluripotency [129]. The frequency of tumor onset has been shown to depend not only on transplanted cell maturation and purity but also on the implantation site and transplantation techniques [130]. This is the reason why it is of such high importance to differentiate hiPSCs in desired and mature cell types before injection. 

### 3.8. Epigenetics in hPSCs

Cell reprogramming is the process of reverting a somatic mature and specialized cell into an iPSC. This process requires the erasure of most of the epigenetic marks established during embryogenesis, organogenesis, and cell differentiation in order to re-establish the self-renewal and pluripotency characteristics of the pluripotent stem cells of the early embryo. Despite the disadvantage in decreasing the reprogramming efficiency when compared to lentiviral/retroviral approaches, non-integrative approaches including episomal plasmid DNA, the Sendai virus, adenovirus, mRNA minicircle vectors, protein transduction, and piggyBac transposon are today used worldwide to obtain hiPSCs. These integration-free methods have been shown to be reliable, and no substantial differences in the quality and safety of cell lines obtained have been highlighted between them [125]. 

The choice of a specific reprogramming method depends on parameters such as final efficiency, reliability, and input cell requirement, as well as on what the final application cells are destined for. However, genome-wide gene expression profiling and DNA methylation pattern analysis need to be regularly carried out to assess the epigenetic stability of hiPSCs whenever possible. Indeed, an incomplete reprogramming of hiPSCs can result in aberrant differentiation and immunogenicity of iPSC-derived cells. It can also be correlated with the hiPSC lineage-specific differentiation potential. Moreover, it has been demonstrated that i) the somatic cell source may play an important role in epigenetic memory [131] but also that ii) the epigenetic signature retention is generally transient, disappearing upon passaging [132]. In fact, the impact of the genomic integrity as well as the epigenetic status in the hiPSC line is not absolute. For example, if differences do not affect cell mutations/differentiation and functions, hiPSC-derived hepatocytes can be used for drug screening, toxicology assays, or the development of bioengineered devices. 

An important question on this subject is “can the epigenetic status contribute to the development or the onset of immunogenicity/tolerance of iPSC-derived tissues?” If it is shown that it can, specific hiPSC lines could be selected to generate hepatic cells with a weak immunogenicity, resulting in an improved cell transplantation outcome for patients [131]. Nonetheless, given the conflicting data available, the mechanisms responsible for the loss/acquisition of immunogenicity are not fully clarified yet. Further work is required to verify whether hiPSC-derived cell epigenetic memory represents an advantage or a real obstacle for clinical application for both autologous and allogeneic hiPSC derivatives [133]. Indeed, the cell’s ability to “be invisible to the immune system” due to an attained or induced loss of immunogenicity may lead to an increase in tumorigenicity. In order to make a quick transition of hiPSC-based therapy to clinics, optimization and new strategies of the reprogramming efficiency need to be achieved with the aim of avoiding genetic and epigenetic abnormalities in the hiPSCs and their derivatives. It will also be valuable to study the immunogenicity of all differentiated cell types, rather than only the undifferentiated hiPSCs. 

The benefits and drawbacks of the different cell types that can be considered for liver therapy and regeneration approaches are summarized in Table 3.

## 4. Conclusions

Straightforward and reproducible, the differentiation protocols of hPSCs available today allow for the generation of most cell-types similar to those of the human body, making this technology very powerful. Due to their continuous proliferation ability, hPSCs constitute the best renewable cell source for research. More than 10 years have passed since hiPSC technology was presented to the scientific community [12]. It has been well demonstrated that they show the same proliferation capacity, plasticity, and pluripotency than hESCs. Moreover, they can be obtained from either healthy individuals or patients by reprogramming adult cell types through a non-invasive procedure. Consequently, they carry the genomic information of the original patient and pave the way for cell-based transplantation therapies and/or human disease modeling, which is a supplementary opportunity with respect to the use of hESCs. On the other hand, there are many limitations to overcome before they can be used in clinics. Despite the tremendous improvements in hiPSC differentiation with the advent of the 3D culture and the organoid generation, HLCs still provide limited functions, and transplantation is currently the only solution to induce cell maturation and the acquisition of a typical adult hepatocyte phenotype. Therefore, if the scientific data recorded from native human tissue samples/animal models and those recorded from organoids are compared, the latter still appears to be very limited. Nevertheless, in this review, hiPSC technologies have been highlighted as a very promising approach to further improve our knowledge on physiology, pathology, and above all liver regeneration, especially using 3D culture systems and organoids. Hopefully, it may not be long before organoids start to be considered as the best in vitro tool to better understand diseases, their diagnoses, and their treatments. hiPSC banks have already been established [139] from both healthy individuals and patients, and the consequent creation of hiPSC-derived organoid biobanks, taking into account human individual diversity, would be particularly useful for drug screening, disease modeling, and cell transplantation. Besides the development of robust and scalable protocols for the differentiation of hiPSCs, many concerns still arise regarding their safety when cell transplants are taken into consideration, for example, the appearance of genetic abnormalities [127,140]. It is important to note that current hiPSC reprogramming and culture management ensure that cells with genetic integrity and stability are obtained. Nonetheless, it is mandatory to check genomic stability on a regular basis, not only before and after the freezing process but also during their regular maintenance [127]. The well-known risk of teratoma development due to the contamination of undifferentiated cells after cell transplantation constitutes another important point for future studies. Procedures able to remove non-differentiated cells from a sample batch generated by differentiation protocols must be set up to decrease the potential risk of tumor formation prior to clinical use. It can certainly be asserted that risks associated with genetic aberrations are overestimated since it has been reported that adult somatic cells contain an incredible number of genetic variations that are not necessarily the onset of cancer [141]. On this basis, it is not surprising that a regulatory guideline has not yet been fully established. One last important comment must be noted. To date, there is no consistent medical experience in the use of hiPSC-based therapeutic approaches. Therefore, it is not easy to foresee how long it will take for hiPSC products to be used for therapeutic liver regeneration approaches or for patient-specific autologous cell treatment. 

## Figures and Tables

**Table 1:** List of the most used methods for the generation of organoids.

Methods for Organoid Generation	
**Scaffold-Based Methods**	Micro-molding Porous 3D scaffolds Poly(N-isopropylacrylamide)-based thermo-responsive surfaces with controllable cell adhesion for cell sheet formation Hepatocyte self-assembly on Primaria dishes
**Scaffold-Free Methods**	Hanging-drop culture Cell culture on non-adhesive surface Centrifugation pellet culture

**Table 2:** Advantages and disadvantages of the discussed current approaches used for liver therapy and regeneration.

	Advantages	Disadvantages
**Orthotopic Liver Transplantation (OLT)**	88% patient survival Clinically defined	Shortage of donors Post-surgery complications Life-long immunosuppressive treatment Organ rejection
**Artificial Liver (AL) Device**	Detoxification ability Bridge patients to OLT	Selective removal/detoxification of toxins Ineffective against encephalopathy Temporary support device
**Cell Transplantation**	Surgical procedure safer and less invasive than OLT Partial correction of liver metabolic disorders	Shortage of cells Transitory improvement of patients’ conditions
**Bio Artificial Liver (BAL) Device**	Improved detoxification ability due to biological components Bridge patients to OLT	Shortage of cells Clinical trials suspended or incomplete Complex set-up and scale-up
**Decellularized/Cellularized** **Liver Scaffolds** **(Pre-Clinical Development)**	Improvement of hepatic cells functions with respect to classic scaffold-based culture approaches Liver-like tissue bio-construction transplantable	Shortage of cells Partial cell repopulation of the scaffolds Slow maturation of the construct Poor viability in pre-clinical studies
**Liver Biofabrication and Bioprinting** **(Pre-Clinical Development)**	Easy scale-up of the 3D liver constructs Improvement of hepatic cell maturations with respect to 3D classic culture approaches	Shortage of cells Slow maturation of the construct Contradictory published data on construct viability

**Table 3:** Summary of advantages and disadvantages of available human cells for liver therapy and regeneration.

Cell	Source	Advantages	Disadvantages	Ref.
**PHHs**	Cadaveric liver Partial hepatectomy	No ethical/political concerns Mature functional cells No risk of teratoma formation Clinically established cells	Immunogenicity Not proliferative in vitro Rapid loss of functionality Not available at large scale Cell aging DNA damage	[23,134]
**FLPs**	Aborted fetus	Highly proliferative Lower immunogenicity than PHHs Transdifferentiation into mature hepatocytes	Ethical concern Low number of cells per fetal liver leading to multiple donors Difficult supply	[135]
**AdLSCs**	Adult liver	Proliferative Bi-potent	Immunogenicity Not available at large scale Cell aging DNA damage	[90,91,92]
**HSCs**	Bone marrow Blood	No ethical concern Highly proliferative Non-invasive collection procedures Abundant supplies (bone marrow) Low viral contamination No risk of teratoma formation Contribution to liver regeneration	Poorly effective: cell fusion with resident hepatocytes/trophic effects Limited number per single cord blood unit (multiple donors) Cell aging DNA damage	[136]
**MSCs**	Bone marrow Umbilical cord Adipose tissue Blood	Highly proliferative Multipotency Immunomodulatory effects Antifibrotic effects	Downregulation of apoptotic genes Downregulation of DNA repair genes Heteroplasmic point mutations Viral transmission Cell aging DNA damage	[137]
**ESCs**	Embryos	Self-renewal Pluripotency	Ethical concern Tumorigenicity Safety concerns (genetic stability) Immunogenicity	[126]
**iPSCs**	Reprogramming of somatic cells	Self-renewal Pluripotency Possibly autologous	Safety concerns Tumorigenicity	[126,138]

PHHs: primary human hepatocytes; FLPs: fetal liver progenitors; AdHLSCs: adult human liver stem cells; HSCs: hematopoietic stem cells; MSCs: mesenchymal stem cells; ESCs: embryonic stem cells; iPSCs: induced pluripotent stem cells.
